# Dynamic LoRA Fine-Tuning of DINOv3 for Multi-Component Pasture Biomass Estimation

**DOI:** 10.3390/s26165285

**Published:** 2026-08-20

**Authors:** Shikha Sen, Nischay Dhankhar, Akram Bayat

**Affiliations:** 1 Khoury College of Computer Sciences, Northeastern University, Boston, MA 02115, USA; a.bayat@northeastern.edu; 2 Nace AI, Palo Alto, CA 94306, USA; nischaydnk@gmail.com

**Keywords:** pasture biomass estimation, Vision Transformer, DINOv3, Low-Rank Adaptation, parameter-efficient fine-tuning, precision agriculture, multi-output regression

## Abstract

Accurate estimation of pasture biomass components from imagery is essential for sustainable grazing management and precision agriculture. Conventional methods such as destructive harvesting, rising plate meters, and remote sensing are limited by scalability, reliability, or the ability to disaggregate biomass by species. We propose a parameter-efficient multi-output regression framework predicting five biomass components (dry green, dry dead, dry clover, green dry matter, and total dry biomass) from high-resolution top-view pasture images. It employs a pretrained DINOv3 Vision Transformer backbone adapted via a dynamic, depth-aware Low-Rank Adaptation (LoRA) strategy, in which the adaptation rank and scaling factor increase exponentially with layer depth: early layers encoding generic visual primitives are minimally perturbed, while deeper layers receive stronger task-specific adaptation. This schedule is effective in low-data regimes, where uniform adaptation or full fine-tuning overfits. To handle rectangular image geometry, each image is split into two square halves processed as a dual-view stream with a contrastive alignment loss. The system ensembles ViT-Large and ViT-Huge backbones with test-time augmentation across five-fold cross-validation. On the CSIRO Image2Biomass benchmark, the full pipeline attains a cross-validated weighted R-squared of 0.81, indicating that depth-aware, parameter-efficient adaptation of large vision models is effective for non-invasive biomass estimation under data scarcity.

## 1. Introduction

Pasture biomass estimation is a critical task in livestock production systems and precision agriculture. Accurate knowledge of the quantity and composition of available feed directly influences decisions on stocking rates, rotational grazing schedules, and pasture recovery management. Grazing systems cover approximately 25% of the global land surface [1], and suboptimal feed management leads to economic losses and environmental degradation, including soil erosion, biodiversity loss, and carbon depletion.

Existing methods for biomass quantification each present significant limitations. The destructive clip-and-weigh technique, while accurate, is labor-intensive and infeasible at scale. Non-destructive proximal sensors such as rising plate meters and capacitance probes offer faster readings but suffer from poor reliability under heterogeneous vegetation conditions and cannot differentiate biomass by species or condition [1]. Satellite-based remote sensing enables broad-scale monitoring but lacks the spatial resolution to capture fine-grained compositional information and still requires manual calibration.

The specific problem addressed in this paper is the simultaneous estimation of five pasture biomass components from high-resolution top-view RGB images under conditions of limited labeled data, non-standard image geometry, and significant distributional variation across geographic regions and seasons. This multi-output regression task is complicated by heavily right-skewed target distributions, zero-inflated clover measurements, and a training set of only 357 annotated images spanning 19 sites across four Australian states.

Large pretrained Vision Transformers (ViTs) such as DINOv3 [2] offer powerful visual representations learned from billions of images. However, deploying these models on small downstream datasets presents a fundamental tension: full fine-tuning updates hundreds of millions of parameters from fewer than 400 labeled examples, making overfitting nearly inevitable, while keeping the backbone entirely frozen forgoes the opportunity to adapt representations to domain-specific patterns such as vegetation density, canopy texture, and species-level discrimination. This tension is especially acute for ViTs, whose self-attention layers learn highly distributed representations that are sensitive to even small perturbations in early layers.

Low-Rank Adaptation (LoRA) [3] offers a principled middle ground by constraining weight updates to low-rank subspaces, dramatically reducing trainable parameter count while preserving the pretrained structure. However, standard LoRA applies a uniform rank across all layers, treating early and late transformer blocks identically. This ignores a well-established property of deep networks: lower layers encode generic, broadly transferable features (edges, textures, color statistics), while deeper layers encode abstract, task-specific representations [4]. In a low-data regime, uniform adaptation allocates capacity to early layers that may need little change and may under-adapt late layers where domain-specific patterns must be learned.

The central question of this paper is: *how should adaptation capacity be distributed across transformer layers when fine-tuning a large pretrained ViT under severe data scarcity?* We propose a dynamic, depth-aware LoRA schedule in which the rank *r* and scaling factor α grow exponentially from the first to the last transformer layer. This provides smooth, continuous control over the degree of adaptation: near-zero perturbation of early layers that encode reusable visual primitives, and progressively stronger specialization in deeper layers where task-relevant compositional patterns must be formed. We observe that this design choice yields a consistent improvement in weighted $R^{2}$ over uniform LoRA, and that it helps preserve pretrained feature quality that otherwise degrades under full fine-tuning and aggressive uniform adaptation in low-data settings.

To instantiate and evaluate this adaptation strategy, we build a complete pipeline for pasture biomass estimation that additionally addresses two practical challenges. First, the dataset images have a 2:1 aspect ratio that conflicts with the square input assumption of pretrained ViTs; we partition each image into two square halves processed as a dual-view stream with a contrastive alignment loss enforcing feature coherence. Second, we introduce a progressive resolution training strategy in which LoRA adapters are trained and merged at successively higher resolutions (384→512→768), which stabilizes the optimization landscape for adapter training. The final system incorporates metadata integration, test time augmentation, and multi backbone ensembling as further supporting components.

The primary contribution of this work is the depth-aware LoRA adaptation scheme and the accompanying empirical analysis demonstrating its advantages over uniform LoRA, full finetuning, and frozen baselines for adapting large ViTs under data scarcity. As secondary contributions, we introduce the dual-view spatial partitioning strategy for handling rectangular imagery and the progressive resolution training curriculum that stabilizes adapter optimization. The complete framework is evaluated on the CSIRO Image2Biomass benchmark [1], where it achieves a cross-validated weighted $R^{2}$ of 0.81.

The remainder of this paper is organized as follows. Section 2 reviews related work on biomass estimation, vision transformers, and parameter-efficient finetuning. Section 3 describes the proposed methodology. Section 4 presents the experimental setup. Section 5 reports quantitative results and ablation studies. Section 6 discusses findings and limitations. Section 7 concludes.

## 2. Related Work

### 2.1. Pasture Biomass Estimation

Traditional approaches to pasture biomass estimation fall into three broad categories: destructive sampling, proximal sensing, and remote sensing. Destructive harvesting remains the gold standard for accuracy but is prohibitively expensive and cannot scale beyond small experimental plots [1]. Proximal sensors such as rising plate meters, capacitance probes, and active optical sensors provide faster non destructive readings, but their accuracy degrades under heterogeneous canopy structures and varying moisture conditions [5]. Satellite and UAV-based remote sensing offers spatial coverage but typically operates at coarse resolution and cannot separate biomass by species or phenological state [6].

More recently, image-based approaches using convolutional neural networks have been explored for biomass estimation from UAV-RGB imagery [7,8]. These works primarily employ standard architectures such as AlexNet and ResNet on aerial orthoimages, treating biomass prediction as a single output regression task. Semi-supervised methods using synthetic data and approximate labels have also been proposed to mitigate the annotation bottleneck [9]. More recently, generative AI assisted preprocessing has been proposed as a complementary route to the same problem: Rana et al. [10] use prompt-guided generative image reconstruction to convert visually heterogeneous vegetation imagery into annotation ready representations that preserve plant morphology while reducing background complexity, facilitating the creation of higher-quality training data for downstream models. This preprocessing stage direction is complementary to the adaptation stage contribution of the present work. However, prior work has largely focused on total biomass rather than component level estimation, and few studies have leveraged modern foundation models or addressed the specific challenges of non standard image geometry in pasture imagery.

Deep learning approaches to pasture and forage biomass estimation remain an active area: Cândido et al. [11] integrate proximal and satellite remote sensing with machine learning for pasture biomass estimation; Azubuike et al. [12] apply transfer learning and stacking ensembles to smartphone imagery across commercial dairy farms; and Mandal [13] recently benchmarks a systematic range of vision foundation model backbones, from EfficientNet-B3 to DINOv3 ViT-Large, together with several cross view fusion mechanisms, on the same CSIRO Image2Biomass dataset used in this work. These studies confirm that CNN- and transformer based deep learning is an active and growing direction for non-destructive biomass estimation, while also underscoring the gap our work addresses: parameter-efficient adaptation of large foundation models under extreme data scarcity (357 training images) and non standard image geometry, conditions that remain largely unaddressed in this recent literature.

### 2.2. Vision Transformers and Self-Supervised Representation Learning

Vision Transformers (ViTs) [14] have emerged as a dominant architecture for visual representation learning, particularly when combined with self-supervised pretraining on large scale datasets. The DINO family of methods [2,15,16] demonstrates that self-supervised ViTs trained with self distillation objectives produce general purpose visual features that transfer effectively to diverse downstream tasks including classification, segmentation, and depth estimation, often without any finetuning. DINOv2 [15] was trained on a curated dataset of 142 million images and showed strong linear probing and *k*-NN performance. DINOv3 [2] scales this further to 1.7 billion images and a 7 billion parameter teacher model, introduces Gram anchoring to address dense feature degradation, and produces improved dense and global representations across over sixty benchmarks. In dense linear probing of the frozen backbone, DINOv3 reaches 55.9 mIoU on ADE20k and 86.6 mIoU on PASCAL VOC, compared to 49.5 and 83.1 for DINOv2 [2], a gap the authors attribute to Gram anchoring’s correction of patch level feature degradation that otherwise emerges under long self-supervised training schedules.

The strength of these pretrained features makes them attractive for domains with limited labeled data, such as agricultural imagery. However, directly deploying frozen features may perform poorly when the target domain differs substantially from the pretraining distribution, as is the case for close range pasture photographs that exhibit fine grained texture variation not well represented in standard image datasets. This motivates the need for controlled adaptation strategies that can bridge the domain gap without degrading the pretrained representation.

### 2.3. Parameter-Efficient Fine Tuning and LoRA for Vision Transformers

Full finetuning of large pretrained models on small downstream datasets is computationally expensive and prone to overfitting. This problem is particularly acute for ViTs, whose self attention layers learn globally distributed representations: perturbations to early layers can propagate through the network and degrade overall feature quality [4]. Parameter-efficient finetuning (PEFT) methods address this by introducing a small number of trainable parameters while keeping the majority of the pretrained model frozen.

LoRA [3] constrains weight updates to low-rank decompositions ΔW=BA, where $B\in R^{d\times r}$, $A\in R^{r\times k}$, and r≪min(d,k). This reduces the parameter count by orders of magnitude while maintaining task performance and introduces no additional inference latency since the low rank updates can be merged into the original weights. Originally proposed for language models, LoRA has since been applied to ViTs for image classification [3], medical imaging [17], and other visual tasks.

However, standard LoRA applies a uniform rank across all layers, which does not account for the well documented functional specialization of transformer layers: lower layers capture generic, transferable features while deeper layers capture increasingly task specific representations [4,18]. In low data settings, uniform adaptation may allocate unnecessary capacity to early layers where the pretrained representation is already appropriate, while under adapting deeper layers where domain specific patterns must be learned. Recent work has explored layer wise learning rate schedules and selective layer unfreezing as alternatives, but these approaches provide coarse, discrete control compared to the smooth, continuous modulation possible with a rank schedule.

Our work introduces a depth aware LoRA scheme that addresses this gap. By increasing the rank and scaling factor with layer depth, we achieve an allocation of adaptation capacity that is better matched to the layer wise feature hierarchy of ViTs. This approach appears especially beneficial when the training set is extremely small, where each degree of freedom in the adaptation must be carefully managed to avoid overfitting.

Despite the availability of strong pretrained vision models and efficient adaptation techniques, there remains a gap in their application to agricultural biomass estimation under the specific constraints of non standard image geometry, limited data, and multi output regression. Our work addresses this gap by combining DINOv3 with dynamic depth-aware LoRA, dual view spatial partitioning, and progressive resolution training.

## 3. Methodology

**Figure 1:**
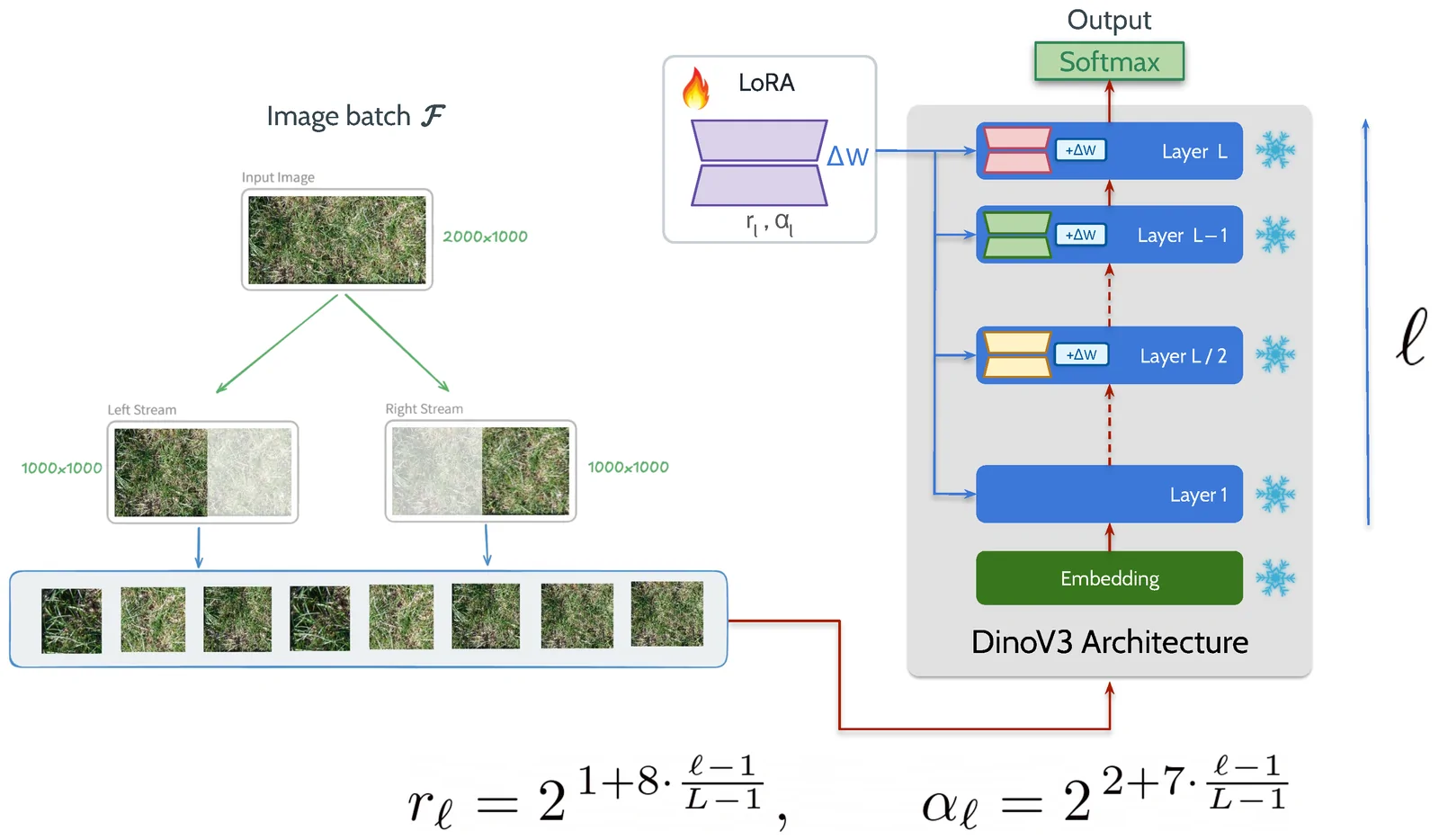
Overview of the proposed pipeline. The input image (2000×1000) is split into left and right square halves, each patchified and passed through a shared DINOv3 backbone with dynamic depth aware LoRA. As shown in the inset, the LoRA rank and scaling factor follow $r_{\ell }=2^{1+8(\ell -1)/(L-1)}$ and $\alpha_{\ell }=2^{2+7(\ell -1)/(L-1)}$, ranging from r=2,α=4 at the first layer to r=512,α=512 at the deepest layer. The pooled embeddings from both streams are concatenated and mapped to five biomass predictions (Grass, Clover, Dry Dead, GDM, Total).

The overall pipeline consists of five components: (1) dual-view spatial partitioning of rectangular images, (2) feature extraction using a DINOv3 backbone with dynamic depth aware LoRA adaptation, (3) contrastive alignment of dual-view embeddings, (4) metadata integration via a dense sub network, and (5) multi output regression with Huber loss. The system is trained using a progressive resolution curriculum and evaluated through five fold stratified group cross validation. An overview of the architecture is shown in Figure 1.

### 3.1. Dual-View Spatial Partitioning

The input images have dimensions of approximately 2000×1000 pixels (width × height), representing a 70×30 cm pasture quadrat captured from above. DINOv3 models are pretrained on square images with a fixed patch size (14 or 16 pixels), and directly resizing a 2:1 aspect ratio image to a square format would compress the horizontal axis and distort spatial relationships between vegetation features.

Instead, each image $I\in R^{3\times H\times W}$ is partitioned along the width dimension into two equal square halves:

(1) $$I^{L}=I\left[:,:,0:W/2\right],\;I^{R}=I\left[:,:,W/2:W\right].$$

Both halves are resized to the target resolution (e.g., 768×768) and processed through the shared DINOv3 backbone in the same batch. The resulting CLS token embeddings $f^{L},f^{R}\in R^{d}$ are concatenated to form the combined visual representation $f_{\mathrm{vis}}=\left[f^{L};f^{R}\right]\in R^{2d}$.

### 3.2. Backbone and Dynamic Depth-Aware LoRA Adaptation

We use DINOv3 ViT-Large (304 M parameters, d=1024, L=24 layers) and DINOv3 ViT-Huge (632 M parameters, d=1280, L=32 layers) as backbone models. Both are initialized from publicly available self-supervised checkpoints and kept largely frozen during training.

#### 3.2.1. The Case for LoRA over Full Fine-Tuning

With only 357 training images and backbone sizes exceeding 300 M parameters, full finetuning creates a severely over parameterized optimization problem. In our experiments, full finetuning of even the last four transformer blocks produced unstable training dynamics and degraded validation performance relative to a frozen baseline (CV $R^{2}$ of 0.70 vs. 0.72). This is consistent with the broader observation that self-supervised ViT representations are holistic: each layer’s output depends on the representations learned in all preceding layers, so perturbation of early layers may disrupt downstream feature representations. LoRA constrains updates to low-rank subspaces, providing plasticity for domain adaptation while limiting the risk of catastrophic forgetting of pretrained features.

#### 3.2.2. Why Uniform LoRA Is Insufficient

Standard LoRA applies the same rank *r* and scaling factor α to every layer. While this is a reasonable default when data is abundant, it becomes less effective in low data settings for two reasons. First, early layers in DINOv3 encode generic visual primitives (edges, local textures, color statistics) that are already well suited to pasture imagery; adapting these layers consumes trainable capacity that could be better allocated elsewhere and risks perturbing features that are already appropriate. Second, deeper layers encode abstract, compositional representations that determine whether the model can distinguish, for example, dead material from green vegetation or clover from grass. These layers stand to benefit most from adaptation, but a uniform rank constrains how much they can change.

#### 3.2.3. Dynamic Depth-Aware Rank Schedule

To address these limitations, we apply LoRA to the query and value projection matrices in each transformer layer’s multi head self attention module with a depth dependent rank schedule. Rather than using a fixed rank *r* across all layers, we increase both the rank and the LoRA scaling factor α exponentially with layer depth:

(2) $$r_{\ell }=r_{\min}\cdot N^{\frac{\ell -1}{L-1}},\;\alpha_{\ell }=\alpha_{\min}\cdot N^{\frac{\ell -1}{L-1}},$$

where ℓ∈{1,…,L} is the layer index, *N* is the growth base, $r_{\min}$ is the minimum rank at the first layer, and $\alpha_{\min}$ is the corresponding minimum scaling factor. In our experiments, we set $r_{\min}=2$ and scale up to $r_{\max}=512$ at the deepest layer, with *N* chosen such that $r_{\min}\cdot N=r_{\max}$.

This schedule produces near-zero perturbation of the earliest layers (rank 2 provides an extremely constrained update subspace), moderate adaptation in mid depth layers, and stronger task specific specialization in the deepest layers where the model must learn to map visual features to biomass quantities. The exponential growth ensures that the transition is smooth rather than abrupt, avoiding the discontinuities introduced by strategies that freeze some layers and fully adapt others.

The depth aware schedule is also parameter efficient in an absolute sense. Because rank grows exponentially, most of the trainable parameters are concentrated in the final layers. The total parameter count (approximately 12.8 M for ViT-Large) is comparable to that of uniform LoRA at r=64 (6.3 M), yet achieves better performance because the parameters are allocated to the layers where adaptation is most needed.

### 3.3. Spatial Alignment Loss

Since the left and right halves originate from the same pasture image, their latent representations should be semantically coherent. To encourage this, we introduce a cosine similarity based alignment loss:

(3) $$L_{\mathrm{align}}=1-\frac{f^{L}\cdot f^{R}}{∥f^{L}{∥}_{2}\;{∥f^{R}∥}_{2}}.$$

This term acts as a regularizer, encouraging the encoder to produce consistent representations across spatial subdivisions of the same sample. It provides an auxiliary training signal that may improve feature quality in low-data regimes where additional supervisory signals are valuable.

### 3.4. Metadata Integration

In addition to visual features, each training sample is accompanied by auxiliary metadata: NDVI (GreenSeeker reading), average sward height (cm), pasture species composition (ordered by biomass), and Australian state. Categorical features (species, state) are mapped to learned embeddings, while continuous features (NDVI, height) are standardized. These metadata features are processed through a small dense sub-network (two fully connected layers with ReLU activation) to produce a metadata embedding $f_{\mathrm{meta}}\in R^{d_{m}}$. The final representation is:

(4) $$f=\left[f_{\mathrm{vis}};f_{\mathrm{meta}}\right]\in R^{2d+d_{m}}.$$

### 3.5. Regression Head and Loss Function

The regression head consists of a two-layer MLP mapping f to five biomass targets. The primary training objective uses Huber loss:

(5) $$L_{\mathrm{task}}=\frac{1}{5}\sum_{j=1}^{5}\ell_{\delta }\left({\hat{y}}_{j}-y_{j}\right),\;\ell_{\delta }\left(r\right)=\left\{\begin{array}{cc} \frac{1}{2}r^{2}, & |r|\leq \delta ,\\ \delta (|r|-\frac{\delta }{2}), & \mathrm{otherwise}. \end{array}\right.$$

The final training objective combines the regression loss and the alignment term:

(6) $$L=L_{\mathrm{task}}+\lambda \;L_{\mathrm{align}},$$

where λ controls the relative weight of the alignment regularizer.

### 3.6. Progressive Resolution Training

Training LoRA adapters directly at high resolution (768×768) on a small dataset can introduce optimization instability. We employ a three stage progressive resolution curriculum:

1. **Stage 1** (384×384): Train LoRA adapters for a full cycle.
2. **Stage 2** (512×512): Merge Stage 1 LoRA weights into the backbone (W←W+BA), attach new LoRA adapters, retrain.
3. **Stage 3** (768×768): Repeat merge-and-retrain at final resolution.

This curriculum is especially relevant for the dynamic LoRA configuration: once a reasonable low resolution adaptation is established, the high rank adapters in deeper layers encounter a better conditioned optimization landscape at higher resolutions.

### 3.7. Data Augmentation and Test Time Augmentation

During training, we apply horizontal and vertical flips (probability 0.5), random 90-degree rotations (probability 0.5), and controlled photometric transformations. At inference, we apply test-time augmentation (TTA):

(7) $$\hat{y}=\frac{1}{T}\sum_{t=1}^{T}f_{\theta }\;\left(I_{\left(t\right)}^{L},I_{\left(t\right)}^{R},m\right).$$

### 3.8. Cross-Validation and Ensembling

We train using five-fold StratifiedGroupKFold with stratification on Australian state and grouping by sampling date. The final system ensembles DINOv3 ViT-Large and ViT-Huge backbones across all folds.

## 4. Experiments

### 4.1. Dataset

We evaluate on the CSIRO Image2Biomass Pasture dataset [1]. The dataset contains 1162 annotated top-view images of temperate pastures collected across 19 locations in four Australian states. Each image captures a 70×30 cm quadrat at approximately 2000×1000 pixel resolution. The training set consists of 357 images, while the test set contains over 800 images with hidden labels. Target distributions are heavily right-skewed with significant zero inflation, particularly for clover.

### 4.2. Evaluation Metric

Performance is evaluated using a globally weighted $R^{2}$ with per-target weights: Dry_Total_g (0.5), GDM_g (0.2), and the remaining three components (0.1 each).

### 4.3. Implementation Details

All models are implemented in PyTorch 2.13.0+cu129 on NVIDIA A100 GPUs. Optimizer: AdamW, initial learning rate $1\times 10^{-4}$, weight decay $3\times 10^{-3}$, cosine schedule with 20-epoch warmup (initial $5\times 10^{-5}$, minimum $1\times 10^{-6}$). EMA is maintained. Dynamic LoRA uses $r_{\min}=2$, $r_{\max}=512$. Alignment loss weight λ=0.1.

## 5. Results

### 5.1. Main Results

Table 1 presents the ablation study, progressively adding components to isolate individual contributions. Public and private leaderboard scores are included as an additional held-out evaluation, since the test labels are not publicly available.

Several patterns emerge. First, full fine-tuning of the last four blocks *degrades* performance below the frozen baseline (CV 0.70 vs. 0.72, private 0.48 vs. 0.52), indicating that unconstrained adaptation is counterproductive under this degree of data scarcity. This motivates the use of parameter-efficient methods.

Second, uniform LoRA recovers a modest improvement over frozen features, but dynamic LoRA provides an additional 2-point CV gain (0.73 → 0.75) and a 3-point gain on both held-out test splits. Among all individual components, the depth-aware adaptation schedule produces the largest single improvement. Notably, the relative benefit of dynamic LoRA over uniform LoRA is preserved across both public and private test splits, suggesting that the improvement reflects generalization rather than overfitting to a particular data partition.

Third, the supporting components (dual-view processing, progressive resolution, metadata integration, and ensembling) each contribute incremental improvements, with the full pipeline reaching a CV score of 0.81.

Weighted $R^{2}$ does not by itself convey absolute error magnitude in physical units, which matters for practical deployment (e.g., kg/ha decisions in stocking-rate planning). Table 2 reports the overall weighted RMSE and MAE for the full pipeline, computed with the same per-target weights and five-fold cross-validation splits as Table 1.

### 5.2. Detailed LoRA Configuration Comparison

Table 3 provides a finer-grained comparison, examining the relationship between adaptation strategy, trainable parameter count, and performance.

Uniform LoRA at r=8 provides no measurable benefit, as the rank is too low to capture task-specific variation. At r=64, a 1-point CV improvement emerges. Increasing to r=256 provides no additional benefit and slightly degrades the private test score (0.53 vs. 0.54), consistent with over-parameterized uniform adaptation beginning to overfit. The dynamic schedule achieves the best performance across all three evaluation settings with a moderate parameter count. This pattern supports the hypothesis that the *distribution* of adaptation capacity across layers matters more than the *total amount* of adaptation capacity.

### 5.3. Effect of Progressive Resolution

Table 4 compares resolution strategies with dynamic LoRA and dual-view processing held constant.

The progressive strategy provides a consistent 1–2 point improvement over direct high-resolution training across all evaluation settings and produces more stable training curves with reduced variance in early-epoch loss.

### 5.4. Benchmark Evaluation

As an additional external evaluation, we submitted the full pipeline to the CSIRO Image2Biomass benchmark [1], which uses a hidden test set of over 800 images. The system achieved a public held-out score of 0.77 and a private held-out score of 0.641. The gap between these scores is analyzed in Section 6.3.

### 5.5. Comparison with Alternative Backbones

To situate the proposed method against conventional and alternative foundation-model backbones, we trained five additional configurations using the same dual view pipeline and five-fold cross-validation protocol: two conventional CNNs (ResNet-50, ConvNeXt-Base), a modern efficient CNN (EfficientNet-B2), a same-scale self-supervised ViT (DINOv2 ViT-Large, 300M parameters), and a full finetuning variant of our own DINOv3 ViT-Large backbone without LoRA. Table 5 reports the results.

Two comparisons are notable. First, DINOv2 at the same 300M-parameter scale as our own backbone falls well short of our result (public $R^{2}$ 0.678 vs. 0.77), indicating that the improvement is not simply a function of backbone size; Section 6 relates this to the Gram anchoring mechanism that distinguishes DINOv3 from DINOv2. Second, and more directly, full fine-tuning of the *same* DINOv3 backbone without LoRA also underperforms our dynamic LoRA configuration on every metric reported (public $R^{2}$ 0.732 vs. 0.77; CV $R^{2}$ 0.761 vs. 0.811), isolating the LoRA adaptation strategy itself, rather than backbone choice alone, as a substantial source of the improvement. All six configurations show a consistent, monotonic ordering across every metric reported.

## 6. Discussion

### 6.1. Depth-Aware Adaptation in Low-Data ViT Settings

The central empirical finding of this work is that the dynamic depth-aware LoRA schedule consistently outperforms uniform LoRA, full fine-tuning, and frozen baselines when adapting large ViTs on a small labeled dataset. We consider three factors that may contribute to this outcome.

**Figure 2:**
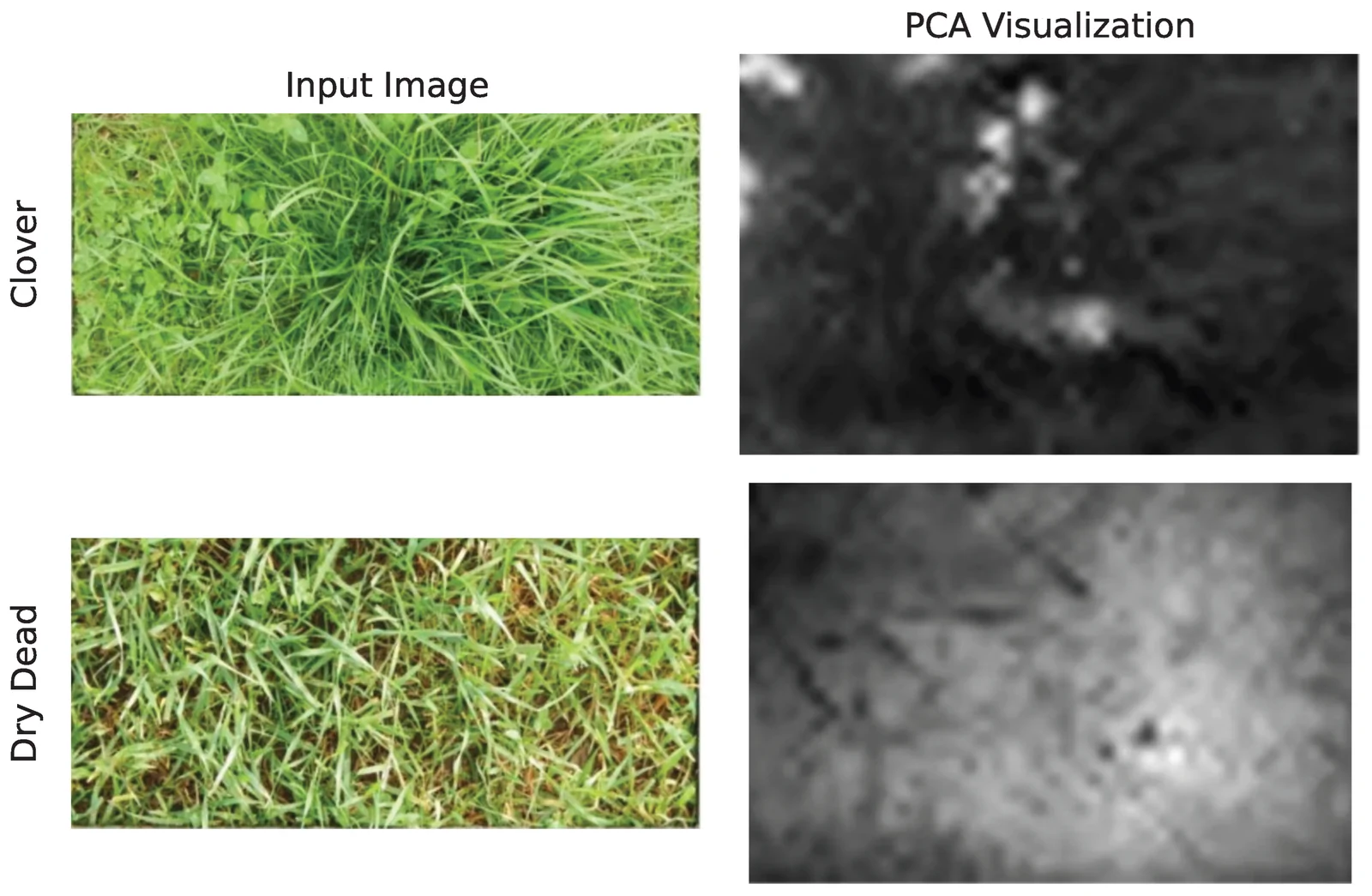
PCA visualization of DINOv3 patch features after dynamic LoRA adaptation. (**Top**): a clover-dominant sample with its first principal component highlighting clover regions (bright areas). (**Bottom**): a dry-dead-dominant sample. These visualizations suggest that the adapted model learns condition-specific feature patterns.

First, ViTs trained with self-supervised objectives such as DINOv3 are known to develop hierarchical feature representations: lower layers encode generic visual primitives, while deeper layers encode abstract, compositional patterns [18]. In pasture imagery, the low-level features needed for edge detection, gradient computation, and local texture statistics are likely well represented in the pretrained model. The domain specific challenge lies in composing these into higher level judgments such as distinguishing dead from green material or estimating clover density. The depth aware schedule concentrates adaptation capacity in the deeper layers where such compositional patterns are formed. Figure 2 provides qualitative evidence that the adapted model produces distinct activation patterns for different biomass compositions.

Second, by constraining early layers to rank 2, the exponential schedule limits the number of trainable parameters that interact with generic features. This may provide an implicit regularization effect, reducing effective model complexity in the region of parameter space most prone to overfitting while still permitting minimal correction when needed. This is a more granular approach than binary freeze-or-adapt strategies that offer no intermediate options. A deeper representational analysis, such as feature covariance or eigenspectrum analysis, could further elucidate this mechanism and is left for future work.

As partial evidence toward this question, Section 5.5 shows that full fine-tuning of the same DINOv3 backbone without LoRA underperforms our dynamic LoRA configuration on every metric reported (CV $R^{2}$ 0.761 vs. 0.811). This demonstrates *that* the depth-aware LoRA strategy outperforms full fine-tuning on an identical backbone, isolating the adaptation strategy itself as a source of the improvement. It does not, however, establish *why* at the level of layer-wise representational change; a mechanistic characterization would require layer-wise analysis of LoRA weight norms and representational similarity (e.g., centered kernel alignment or cosine similarity between pretrained and adapted features) between early and late transformer blocks. We were unable to complete this analysis for the present study and identify it as a concrete direction for follow-up work.

Third, the progressive resolution training appears to interact favorably with the depth-aware schedule. The merge-and-retrain procedure at each resolution stage allows each stage to build on adapted features from the previous stage, which may particularly benefit the high-rank adapters in deeper layers by providing them a better-conditioned optimization landscape.

For practitioners adapting large ViTs to small scientific or agricultural datasets, these results suggest that a depth-aware LoRA schedule merits consideration over uniform LoRA or partial fine-tuning. The observed performance gain is achieved at no additional inference cost, since all LoRA weights are merged into the backbone after training.

### 6.2. Dual-View Processing

The 2-point improvement from dual view processing with alignment loss indicates that preserving the pretrained model’s spatial assumptions is beneficial for downstream performance. The single stream baseline, which resizes the full 2000×1000 image to 768×768, compresses the horizontal dimension by approximately 2.6×, distorting vegetation textures and spatial patterns. The dual view approach avoids this distortion and additionally provides the alignment loss as an auxiliary regularization signal.

### 6.3. Analysis of the Public-Private Test Gap

The model exhibited a gap between public (0.77) and private (0.641) held-out test performance. This pattern was common across submissions to the benchmark, with many high scoring public configurations experiencing comparable drops. We analyze this gap along two dimensions.

First, the ratio of private to public $R^{2}$ is approximately 0.82±0.02 across all ablation configurations in Table 1. This consistency suggests that the gap reflects a distributional property of the data split rather than a failure mode specific to any individual component. Notably, dynamic LoRA outperforms uniform LoRA on *both* test splits (public: 0.68 vs. 0.65; private: 0.57 vs. 0.54), indicating that the relative benefit of depth-aware adaptation is robust to the observed distribution shift.

Second, the CSIRO dataset spans four Australian states with distinct climatic regimes, and analysis of the training metadata reveals that species composition varies substantially across states (e.g., Victorian sites are predominantly perennial ryegrass, while Western Australian sites contain more annual species). If the two test splits sample different geographic or seasonal strata, the resulting distributional mismatch would produce a performance gap without necessarily implying overfitting.

### 6.4. Limitations

Several limitations should be noted. First, the training set is small (357 images), which constrains learnable patterns and contributes to the generalization gap. Second, the method relies on metadata features that may not always be available in deployment settings. Third, the dynamic LoRA schedule introduces additional hyperparameters ($r_{\min}$, $r_{\max}$, *N*), though we found the method to be relatively robust to moderate variations in a preliminary sensitivity analysis. Fourth, processing two views per image doubles inference FLOPs, though this remains modest compared to full fine-tuning. Fifth, our evaluation is limited to a single dataset and application domain; further investigation on other low-data visual regression tasks would strengthen the generality of the findings. Sixth, while Section 5.5 shows that dynamic LoRA outperforms full fine-tuning of the same backbone, a layer-wise mechanistic analysis (e.g., LoRA weight-norm profiles or representational similarity between pretrained and adapted features) that would explain *why* the depth-aware schedule is effective was not completed for this study and is left for future work.

## 7. Conclusions

We have presented a parameter efficient framework for multi component pasture biomass estimation from top view imagery. The primary methodological contribution is a dynamic depth aware LoRA adaptation strategy for DINOv3 Vision Transformers, in which the rank and scaling factor increase exponentially with layer depth. This design allocates minimal adaptation capacity to early layers, where pretrained generic features are already appropriate, and progressively stronger adaptation to deeper layers, where task-specific compositional patterns must be formed. In our experiments, this schedule consistently outperformed uniform LoRA, full finetuning, and frozen baselines on both cross validation and held out test evaluation, suggesting that principled allocation of adaptation capacity across transformer layers is an important design consideration for parameter efficient finetuning under data scarcity.

The complete framework additionally incorporates dual view spatial partitioning to accommodate non standard image geometry, progressive resolution training to stabilize adapter optimization, and metadata integration to complement visual features. Together, these components yield a cross validated weighted $R^{2}$ of 0.81 on the CSIRO Image2Biomass benchmark while adapting fewer than 13 M parameters out of a 304 M parameter backbone.

More broadly, these results indicate that the question of *how* to distribute adaptation capacity across layers deserves attention alongside the established question of *how much* total adaptation to apply. Future work could explore the depth-aware schedule on other low data vision tasks, extend it to other PEFT methods such as adapters and prefix tuning, incorporate temporal or spectral data for field scale deployment, and provide deeper theoretical grounding through representational analysis of layer wise feature dynamics during adaptation.

## Figures and Tables

**Table 1:** Ablation study on the CSIRO Image2Biomass dataset. All models use DINOv3 ViT-Large unless noted. Pub. and Priv. denote public and private held-out test weighted $R^{2}$.

Configuration	CV	Pub.	Priv.
Frozen DINOv3 (head only)	0.72	0.63	0.52
Full fine-tune (last 4 blocks)	0.70	0.59	0.48
Uniform LoRA (r=64)	0.73	0.65	0.54
Dynamic LoRA	0.75	0.68	0.57
+ Dual-View + Align. Loss	0.77	0.72	0.59
+ Progressive Resolution	0.78	0.73	0.60
+ Metadata	0.79	0.74	0.61
+ TTA + Ensemble (L+H)	**0.81**	**0.77**	**0.641**

**Table 2:** Overall weighted error magnitude for the full pipeline (five-fold CV), reported alongside weighted $R^{2}$ for interpretability.

Metric	Value
Weighted RMSE (CV)	10.23 g
Weighted MAE (CV)	7.09 g
Weighted $R^{2}$ (CV)	0.811

**Table 3:** Comparison of LoRA configurations on DINOv3 ViT-Large.

Configuration	Params	CV	Pub.	Priv.
No LoRA (frozen)	0.1 M	0.72	0.63	0.52
Uniform r=8	0.8 M	0.72	0.63	0.52
Uniform r=64	6.3 M	0.73	0.65	0.54
Uniform r=256	25.2 M	0.73	0.64	0.53
Dynamic (r:2→512)	12.8 M	**0.75**	**0.68**	**0.57**

**Table 4:** Effect of training resolution strategy with dynamic LoRA on DINOv3 ViT-Large.

Strategy	CV	Pub.	Priv.
Single: 384×384	0.74	0.67	0.55
Single: 768×768	0.76	0.70	0.57
Progressive: 384→512→768	**0.77**	**0.72**	**0.59**

**Table 5:** Comparison with alternative backbone architectures, all trained with the same dual-view pipeline and five-fold cross-validation protocol as the proposed method.

Backbone	Params	CV	CV RMSE	CV MAE	Pub.
ConvNeXt-Base (FT)	89 M	0.641	21.51	13.16	0.550
ResNet-50 (FT)	24 M	0.650	18.01	11.54	0.572
EfficientNet-B2 (FT)	9.2 M	0.686	17.26	10.10	0.601
DINOv2 ViT-L (FT)	300 M	0.729	16.01	8.31	0.678
DINOv3 ViT-L (FT, no LoRA)	300 M	0.761	11.11	7.40	0.732
DINOv3 ViT-L + Dynamic LoRA (ours)	300 M (12.8 M tr.)	**0.811**	**10.23**	**7.07**	**0.77**

## Data Availability

The data analyzed in this study are openly available. Dataset: CSIRO Image2Biomass pasture dataset (creator: Commonwealth Scientific and Industrial Research Organisation (CSIRO); described in [1]), published and hosted by Kaggle and available at https://www.kaggle.com/competitions/csiro-biomass/data (accessed on 4 July 2026). No new data were created in this study.

## Abbreviations

The following abbreviations are used in this manuscript:

ViT	Vision Transformer.
LoRA	Low-Rank Adaptation.
PEFT	Parameter-Efficient Fine-Tuning.
CV	Cross-Validation.
TTA	Test-Time Augmentation.
NDVI	Normalized Difference Vegetation Index.
GDM	Green Dry Matter.
MLP	Multi-Layer Perceptron.
EMA	Exponential Moving Average.
